# Editorial: Policy issues and perspectives in referrals and access to quality health care services

**DOI:** 10.3389/frhs.2023.1323442

**Published:** 2023-11-30

**Authors:** D. O. Akeju, B. O. Okusanya, U. V. Ukah, S. O. Orimaye, O. Dirisu

**Affiliations:** ^1^ Department of Sociology, University of Lagos, Lagos, Nigeria; ^2^ College of Medicine, Lagos University Teaching Hospital, University of Lagos, Lagos, Nigeria; ^3^ Pregnancy and Child Research Centre, HealthPartners, Bloomington, MN, United States; ^4^ College of Global Population Health, University of Health Sciences and Pharmacy in St. Louis, St. Louis, MO, United States; ^5^ Nigerian Economic Summit Group, Abuja, Nigeria

**Keywords:** health policy, referral, quality healthcare, healthcare services, access to healthcare

**Editorial on the Research Topic** Policy issues and perspectives in referrals and access to quality health care services

## Introduction

This editorial synthesizes findings from the 4 articles submitted under this Research Topic, with specific focus on different aspects of referral services and other policy issues. These articles and other similar articles emphasize the relevance of referral to quality healthcare and also highlight the core barriers to an effective referral system. The aim is to draw the attention of policymakers on this important but relegated aspect of healthcare and to provide a succinct summary of the key findings from these articles, underscoring their importance in enhancing our understanding of how referral services can impact on access to healthcare. In this editorial, we reflect on a chain reaction of multiple factors impeding effective referral services and access to quality health.

## The significance of referral and its influence on health outcomes

Referral is a system that guides the process of transferring and sharing the responsibility of a patient's care between healthcare professionals and from one level of care to another (1, 2). It is one of the core aspects of healthcare services that is significantly related to the Sustainable Development Goals (SDG 3). As evidenced in the literature, delay in referral or wrong referral can lead to poor health outcomes (3). Therefore, it is important to consider the challenges associated with referral services, the policies guiding it, and the environment within which these policies are implemented. Policy frameworks that guide the implementation of referral services are influenced by contexts and therefore outcomes of referral services differ significantly across contexts as do the challenges associated with them. In spite of the significance and relevance of referral systems to enabling better health outcomes, it has not received concerted global attention from policy makers and key players in the health sector. Hence, it is imperative to examine policy issues that impact on the execution of referral services and their implications on the quality of healthcare services.

## Effective referral system

For many decades, the literature has shown that the lack of an effective referral system is a major challenge to quality healthcare services (2–7). One way the health systems can effectively respond to chronic and infectious diseases and emergent health matters, such as hemorrhage, obstructed labor, and hypertension is by establishing quick, effective and responsive referral systems (7–9). By systems, we refer to a set of practices which synchronize patient data transmission from one physician to physician, physician to patient. The practices encompass all the domains of the health systems that are relevant to referrals, such as transportation, follow-up, monitoring and evaluation (1, 5). These domains manifest differently in different social, political, and cultural contexts.

## Resource constraints, referrals and quality of care

Human resource constraints at both lower and higher levels of care place the healthcare systems in most of Africa and Asia at huge disadvantage (10). The brain drain of health professionals in Africa and Asia has reduced the doctor-patient ratio far below what is acceptable (11), with the consequence of a loss of confidence in service users who patronize higher levels of care when referred (12). As research has shown in Uganda and other low and middle-income countries (LMICs), capacity related barriers negatively impact access to quality healthcare (Muwanguzi et al.). This might partly account for why many pregnant women in Ethiopia adopt a self-referral strategy while seeking healthcare (Eshetie et al.).

## Barriers to referrals and health systems resilience

Annually, millions of preventable maternal and child deaths occur across the globe as a result of poor referral services (3, 5, 13). In India, as in many other LMICs, the most common referrals, as observed by Das et al. were due to pregnancy-induced hypertension or eclampsia, previous caesarean section, fetal distress and oligohydramnios. These underscore the importance of referral services in LMICs. Paradoxically, in Africa and in Asia with high fertility rates, there is a huge challenge in referral services (14, 15). Apart from poverty, which is a significant barrier to accepting or completing a referral process at higher levels in LMICs, socio-cultural issues have been implicated. Thus, barriers to effective referral services have consistently remained, especially in Africa and Asia (3, 5, 7, 8, 16). In one of the articles in this collection, capacity related barriers to cognitive impairment screening such as chronic understaffing, lack of training/skills, lack of knowledge and awareness in screening, among others, were identified as core challenges to access quality healthcare by older adults (Muwanguzi et al.). In addition, antenatal follow-ups, clients' knowledge of the referral system, and mode of transportation, cost of care, and proximity of patients to health facility, were factors significantly associated with the poor referral practice (2, 9, Muwanguzi et al., Eshetie et al., Visscher et al.).

The uncertainties and challenges imposed by the recent COVID-19 pandemic show the vulnerability of the health systems to sudden disruption at a global scale (17, 18). A study showed that during a pandemic, people with poor health are most likely to experience postponed healthcare and negative health consequences and more often will decide to forgo healthcare (Visscher et al.). The COVID-19 pandemic experience has shown that access to quality healthcare is predicated on the availability of skilled healthcare workers and resilient health infrastructure (Visscher et al.). Amidst these challenges, integrating e-referral with telemedicine and remote patient monitoring could be a viable option for expanding access through effective referral (19).

## Addressing challenges involving referral services

To address healthcare referral and other policy issues affecting access to quality healthcare, this collection and evidence from other scientific articles have emphasized the need for adequate transportation facilities to enhance emergent referrals (1, 8, 20, 21). Others have emphasized the need for follow up with patients and improved feedback systems, especially those requiring obstetric care at higher levels (Eshetie et al., Das et al., Visscher et al.) or needing HIV-related services (22). Furthermore, providing a policy framework including referral protocol, referral service directory, and guidelines can remove some of the barriers to referral services (8). A successfully executed referral enables the patient to access the service for which they were referred and the services received are confirmed through a referral feedback loop system (2).

The advantages and the downsides of an integrated information system in closing the gaps in effective referral systems have been reported (6, 9). For example, access to telemedicine and remote patient monitoring for hard-to-reach populations could improve referral systems (6, 9, 19). In spite of known challenges, a policy framework that integrates all domains of referral into a system driven by technology will potentially facilitate progress, especially in LMICs. A triage system driven by technology can also enhance referral of emergent cases and health complications. An integrated approach involving the community, policy makers, stakeholders and government institutions or agencies is critical to achieving an effective referral system and creating access to quality health.
